# The influence of stress hyperglycemia on consciousness disturbance and short- and long-term outcomes in stroke patients without documented diabetes: Differences between ischemic and hemorrhagic stroke

**DOI:** 10.1371/journal.pone.0331077

**Published:** 2025-08-26

**Authors:** Jing Feng, Chang He, Hongbo San, Mengsi Zhan, Hongyu Zhang, Jianheng Gu, Hongqian Fu, Jiahao Yao, Lang Li, Xuefeng Wang

**Affiliations:** 1 Department of Neurosurgery, The Fourth Affiliated Hospital of Harbin Medical University, Harbin, China; 2 Department of neurology, People’s Hospital of Ningxiang City, Hunan, China; Fondazione Policlinico Universitario Agostino Gemelli IRCCS, ITALY

## Abstract

**Background:**

Although hyperglycemia is a well-known prognostic factor in diabetic stroke patients, its impact on outcomes in individuals without documented diabetes remains insufficiently explored. This study aimed to investigate the relationship between stress-induced hyperglycemia and both short- and long-term mortality, as well as impaired consciousness, in patients without documented diabetes with ischemic or hemorrhagic stroke.

**Methods:**

A retrospective cohort study was conducted using the MIMIC-IV (v3.1) database, including ICU-admitted patients without documented diabetes (ischemic or hemorrhagic stroke identified via ICD-9/10 codes). Exclusion criteria were: age < 18 years, missing glucose values, and severe impairment of consciousness upon ICU admission, defined as a Glasgow Coma Scale (GCS) score < 8. After applying these criteria, 4,151 patients were enrolled. Propensity score matching (PSM) was performed to balance baseline covariates between the hyperglycemic (HG) and non-hyperglycemic (Non-HG) groups. Cox proportional hazards models and Kaplan-Meier survival curves were used to assess the impact of hyperglycemia on mortality and neurological outcomes. Then subgroup analysis was conducted.

**Results:**

Following PSM, 1,170 matched pairs were analyzed. In patients with hemorrhagic stroke (HS), stress hyperglycemia was associated with an increased 1-year mortality risk (HR = 0.71, p = 0.029). Among ischemic stroke (IS) patients, hyperglycemia independently predicted both in-hospital and 1-year mortality (HR range: 0.65–0.79, p < 0.001). Kaplan-Meier analyses showed significantly poorer survival in hyperglycemic patients. Subgroup analysis demonstrated that stress hyperglycemia was particularly detrimental in HS patients with heart failure, pneumonia, vasopressin use, or severe neurological impairment (GCS < 8 with heart failure). In IS patients, hyperglycemia was significantly associated with worse outcomes in those with advanced age, coronary artery disease, COPD, pneumonia, renal failure, or norepinephrine treatment.

**Conclusion:**

Stress hyperglycemia independently predicts poor outcomes in stroke without documented diabetes, especially in ischemic subtypes. These findings highlight the importance of early glycemic assessment and tailored management strategies. Further research is warranted to validate causal mechanisms. In hemorrhagic stroke, its impact appears more delayed, affecting long-term prognosis. These findings support the need for early recognition and tailored glycemic control strategies based on stroke subtype.

## 1. Introduction

Stroke continues to pose a significant global health challenge, affecting approximately 50 million individuals each year, with a lifetime risk nearing 25% [1]. Ischemic stroke—the most common subtype—primarily results from arterial occlusion or venous thrombosis [2]. In contrast, hemorrhagic strokes, accounting for 10% to 40% of all cases, are typically caused by vascular rupture or aneurysmal bleeding and include intracerebral and subarachnoid hemorrhages [3,4].

Stress-induced hyperglycemia worsens brain injury through various mechanisms, including endothelial dysfunction, oxidative stress, disruption of the blood–brain barrier (BBB), and increased inflammatory responses [5,6]. It has been reported in 30%–40% of ischemic stroke (IS) cases and 43%–59% of hemorrhagic stroke (HS) cases [7]. This condition arises from acute stress responses that trigger elevations in glucocorticoids, catecholamines, and proinflammatory cytokines (e.g., IL-6 and TNF-α), ultimately promoting insulin resistance and enhanced hepatic glucose production [8,9]. In the context of stroke, hyperglycemia has been associated with cerebral edema, infarct expansion, BBB disruption, and a higher risk of hemorrhagic transformation [10]. In IS, it may hinder collateral perfusion and reperfusion processes, whereas in HS, it may facilitate hematoma expansion and secondary inflammatory responses [11,12]. Collectively, these effects contribute to early neurological decline and heightened short-term mortality.

Although stress hyperglycemia (SHG) has been extensively investigated in diabetic stroke populations, its prognostic significance in patients without documented diabetes remains unclear. Recent studies suggest that even transient elevations in blood glucose may adversely affect outcomes, leading to larger infarct volumes, greater neurological impairment, and higher mortality rates [13,14]. Therefore, this study aimed to assess the relationship between SHG and both short- and long-term mortality, as well as impaired consciousness, in patients with either ischemic or hemorrhagic stroke. Additionally, we sought to identify vulnerable subpopulations and provide evidence to guide individualized glycemic management strategies in acute stroke care.

## 2. Methods

### 2.1. Data sources and ethical considerations

Clinical data were obtained from the Medical Information Mart for Intensive Care IV (MIMIC-IV, version 3.1), an updated and expanded version of MIMIC-III [15]. The MIMIC-IV database is publicly available and includes de-identified health records from over 65,000 ICU admissions and more than 200,000 emergency or ICU encounters at Beth Israel Deaconess Medical Center (BIDMC) between 2008 and 2022. Use of the MIMIC-IV database was approved by the institutional review boards of BIDMC and the Massachusetts Institute of Technology (MIT), with a waiver of informed consent due to anonymization. Ethical approval was waived by the Institutional Review Board (IRB) because the study used de-identified data from the publicly available MIMIC-IV database and did not involve direct contact with human participants. The author (Jing Feng) was granted access to the database after completing the Collaborative Institutional Training Initiative (CITI) program (Certificate No. 66611501). The data were accessed for research purposes between October 11, 2024, and November 20, 2024. The authors did not have access to any information that could identify individual participants during or after data collection, as all personally identifiable information had been removed from the dataset. The study was conducted in accordance with the Declaration of Helsinki.

### 2.2. Study populations and variable extraction

#### 2.2.1. Inclusion and exclusion criteria.

**Fig 1:**
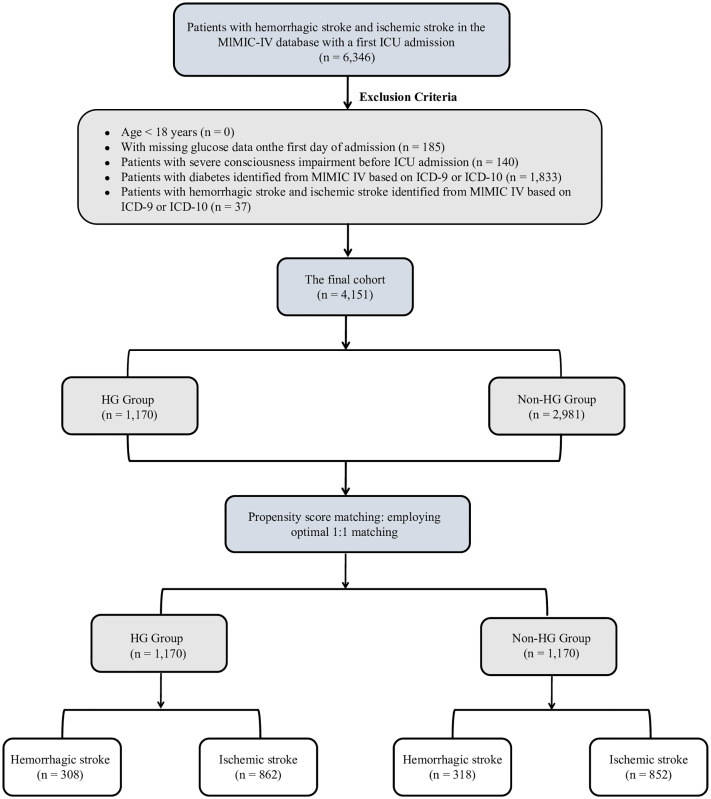
Flowchart illustrating the screening process of critically ill patients with stroke from the MIMIC-IV database.

Patients classified as having ischemic stroke (IS) or hemorrhagic stroke (HS) under ICD-9 or ICD-10 codes were eligible for inclusion. Exclusion criteria were as follows: (1) age under 18 years; (2) no ICU admission; (3) missing blood glucose measurements; (4) a diagnosis of diabetes mellitus; (5) severely impaired level of consciousness at ICU admission, defined as a Glasgow Coma Scale (GCS) score <8; or (6) patients with both ischemic stroke and hemorrhagic stroke recorded as separate diagnoses were excluded. Hemorrhagic transformation of ischemic stroke was not systematically identified in the database and therefore was not excluded. Diabetes mellitus was excluded based on structured ICD codes and documented comorbidities prior to ICU admission. Although HbA1c is a useful marker for chronic hyperglycemia, it was not consistently available in this ICU cohort and could not be applied as a standard exclusion criterion. In cases of repeated admissions, data from the initial admission were used. A final cohort of 4,151 patients fulfilled the eligibility requirements and were enrolled in the research (Fig 1).

#### 2.2.2. Definition of hyperglycemia and blood glucose collection.

For each patient, the baseline glucose value was defined as the first blood glucose measurement obtained within 24 hours of ICU admission. Only this first recorded value was used for classification and subsequent analysis. These values were not collected under standardized fasting conditions and were generally recorded as part of emergency or routine assessments shortly after ICU entry. As such, they are considered random, non-fasting glucose values reflective of acute physiological stress. Patients were classified into hyperglycemia (HG, > 140 mg/dL) and non-hyperglycemia (non-HG, ≤ 140 mg/dL) groups accordingly.

#### 2.2.3. Stroke subtype stratification and ICU context.

Given the substantial heterogeneity between IS and HS in terms of pathophysiology and prognosis, all subsequent analyses were performed separately for each stroke subtype. In the MIMIC-IV database, all included stroke patients were admitted to the ICU due to acute neurological deterioration, respiratory failure, or other critical complications requiring intensive monitoring and intervention. This reflects the institutional practice at Beth Israel Deaconess Medical Center, where severely ill stroke patients are often managed in the ICU rather than in a stroke unit.

#### 2.2.4. Variable extraction and missing data handling.

We extracted all variables from the MIMIC-IV database using SQL in PostgreSQL, covering five major categories [16]: demographics, vital signs, comorbidities, laboratory parameters, and clinical scoring systems. To address missing data, values in the dataset were imputed using the random forest method. Missing values in the matching variables mainly occurred because the data were randomly absent. Patient characteristics, predictor variables, and outcome measures were all extracted using structured SQL queries (S1 Table in S1 File).

### 2.3. Clinical outcomes

The primary outcomes were in-hospital mortality and a significant decline in consciousness (GCS < 8) within 30 days of admission. Secondary outcomes included all-cause mortality at 30, 90, 180 days, and 1 year.

### 2.4. Statistical analysis

Continuous variables were reported as means ± standard deviations, whereas categorical variables were expressed as counts and percentages. Comparisons of continuous variables across groups were performed using the t-test or analysis of variance. Fisher’s exact test or the chi-square test was used to compare categorical variables. Patients were categorized according to blood glucose levels as follows: non-hyperglycemia group (non-HG) (≤ 140 mg/dL(7.8 mmol/L)) and hyperglycemia group (HG) (> 140 mg/dL) [17,18]. Propensity score matching (PSM) was utilized to minimize selection bias between the hyperglycemic (HG) and non-hyperglycemic (Non-HG) groups. Propensity scores were estimated using logistic regression, incorporating the following covariates: age, sex, stroke type (ischemic vs. hemorrhagic), coronary artery disease, heart failure, pneumonia, renal failure, norepinephrine use, vasopressin use, GCS score on admission, and SOFA score. Matching was performed using 1:1 optimal matching without replacement and a caliper width of 0.2 standard deviations of the logit of the propensity score, implemented via the MatchIt package in R. Ultimately, a total of 1,170 matched pairs were successfully generated [19].

Univariable and multivariable Cox proportional hazards models were used to evaluate the association between stress hyperglycemia and mortality outcomes. Three hierarchical models were constructed, adjusting for potential confounders. Results are reported as hazard ratios (HRs) with 95% confidence intervals (CIs). Kaplan–Meier survival curves and log-rank tests were applied to compare survival between the hyperglycemic and non-hyperglycemic groups at in-hospital, 30-day, 90-day, 180-day, and 1-year intervals. Additional survival analysis was conducted for patients with severe impaired consciousness (GCS < 8).

Subgroup analyses were performed across key clinical and demographic variables, including age (> 54.5 vs. ≤ 54.5 years based on cohort median), sex, comorbidities (hypertension, coronary artery disease, heart failure, COPD, pneumonia, renal/kidney disease), and treatments (mannitol, norepinephrine, vasopressin, invasive ventilation, surgery). Two-sided *P*-values < 0.05 were considered statistically significant. All analyses were conducted using R software (version 4.2.2). A sensitivity analysis was performed including all hyperglycemic stroke patients, stratified by documented diabetes status, to assess the robustness of the main results.

## 3. Results

### 3.1. Baseline characteristics

A total of 4,151 stroke patients without documented diabetes were included and divided into hyperglycemic (HG, N = 1,170) and non-hyperglycemic (non-HG, N = 2,981) groups. Significant baseline differences were observed between groups within each stroke subtype (hemorrhagic or ischemic; Table 1). To reduce confounding, 1:1 optimal propensity score matching (PSM) was performed separately within each stroke subtype, yielding 1,170 matched pairs. Covariate balance was assessed using absolute standardized differences (ASDs), with values <0.1 indicating adequate balance. These results are illustrated in S1 Fig in S2 File.

**Table 1:** P-values compare HG vs non-HG patients within each stroke subtype (hemorrhagic or ischemic).

Characteristic	Stroke (hemorrhagic stroke), N = 626			Stroke (ischemic stroke), N = 1714		
	HG Group N = 318	Non-HG Group N = 319	p-value^1^	HG Group N = 852	Non-HG Group N = 862	p-value^1^
Age	73 ± 13	73 ± 14	0.924	65 ± 16	63 ± 17	0.06
Sex			0.423			0.837
Male	115 (36.2%)	115 (36.3%)		449 (52.7%)	450 (52.2%)	
Female	203 (63.8%)	203 (63.9%)		403 (47.3%)	412 (47.8%)	
Weight	77 ± 21	77 ± 22	0.819	78 ± 21	77 ± 29	0.324
Temperature	36.73 ± 0.70	36.73 ± 0.71	0.352	36.87 ± 1.82	36.92 ± 1.31	0.546
Respiratory rate	19.9 ± 5.4	19.9 ± 5.5	0.357	19.1 ± 5.0	18.2 ± 4.5	<0.001
Heart rate	84 ± 19	84 ± 20	0.049	83 ± 19	78 ± 17	<0.001
Systolic Blood Pressure	114 ± 22	114 ± 23	0.759	125 ± 22	124 ± 20	0.252
Diastolic Blood Pressure	63 ± 16	63 ± 17	0.012	69 ± 16	69 ± 15	0.379
Mean Blood Pressure	74 ± 16	74 ± 17	0.364	83 ± 16	83 ± 16	0.389
SpO2	96.1 ± 6.8	96.1 ± 6.9	0.301	97.60 ± 3.93	97.37 ± 2.64	0.157
Hypertension			0.535			0.475
No	177 (55.7%)	177 (55.8%)		363 (42.6%)	382 (44.3%)	
Yes	141 (44.3%)	141 (44.4%)		489 (57.4%)	480 (55.7%)	
Coronary			0.79			0.399
No	135 (42.5%)	135 (42.6%)		748 (87.8%)	768 (89.1%)	
Yes	183 (57.5%)	183 (57.6%)		104 (12.2%)	94 (10.9%)	
Heart failure			0.258			0.507
No	137 (43.1%)	137 (43.2%)		770 (90.4%)	787 (91.3%)	
Yes	181 (56.9%)	181 (56.10%)		82 (9.6%)	75 (8.7%)	
COPD			0.658			0.549
No	300 (94.3%)	300 (94.4%)		845 (99.2%)	857 (99.4%)	
Yes	18 (5.7%)	18 (5.8%)		7 (0.8%)	5 (0.6%)	
Pneumonitis			0.012			0.001
No	216 (67.9%)	216 (67.10%)		620 (72.8%)	685 (79.5%)	
Yes	102 (32.1%)	102 (32.2%)		232 (27.2%)	177 (20.5%)	
Renal failure			0.677			0.039
No	189 (59.4%)	189 (59.5%)		721 (84.6%)	759 (88.1%)	
Yes	129 (40.6%)	129 (40.7%)		131 (15.4%)	103 (11.9%)	
Kidney disease			0.295			0.279
No	230 (72.3%)	230 (72.4%)		796 (93.4%)	816 (94.7%)	
Yes	88 (27.7%)	88 (27.8%)		56 (6.6%)	46 (5.3%)	
Hepatitis			0.643			0.863
No	304 (95.6%)	304 (95.7%)		817 (95.9%)	828 (96.1%)	
Yes	14 (4.4%)	14 (4.5%)		35 (4.1%)	34 (3.9%)	
Malignancy			0.961			0.812
No	239 (75.2%)	239 (75.3%)		705 (82.7%)	717 (83.2%)	
Yes	79 (24.8%)	79 (24.9%)		147 (17.3%)	145 (16.8%)	
WBC count	12.6 ± 6.6	12.6 ± 6.7	0.05	13.3 ± 10.7	11.2 ± 8.8	<0.001
RBC count	3.65 ± 0.73	3.65 ± 0.74	<0.001	3.95 ± 0.73	3.98 ± 0.69	0.345
Platelet count	205 ± 102	205 ± 103	0.127	219 ± 90	216 ± 86	0.445
Hemoglobin	11.13 ± 2.14	11.13 ± 2.15	<0.001	11.93 ± 2.08	12.05 ± 2.02	0.237
HCT	34 ± 6	34 ± 7	<0.001	35.7 ± 5.9	36.2 ± 5.7	0.08
MCH	30.55 ± 2.42	30.55 ± 2.43	0.469	30.49 ± 2.29	30.43 ± 2.40	0.606
MCHC	33.00 ± 1.54	33.00 ± 1.55	0.348	33.45 ± 1.44	33.29 ± 1.39	0.023
Sodium	137.6 ± 5.4	137.6 ± 5.5	0.648	138.7 ± 4.7	139.3 ± 4.4	0.009
Potassium	4.23 ± 0.71	4.23 ± 0.72	0.413	3.98 ± 0.64	3.92 ± 0.56	0.046
Calcium	8.32 ± 0.88	8.32 ± 0.89	0.668	8.60 ± 0.75	8.66 ± 0.75	0.104
Chloride	104 ± 6	104 ± 7	0.464	104.2 ± 5.4	104.6 ± 5.1	0.148
phosphorus	3.90 ± 1.52	3.90 ± 1.53	0.392	3.35 ± 1.16	3.33 ± 0.84	0.763
Creatinine	1.55 ± 1.34	1.55 ± 1.35	0.267	0.99 ± 0.67	0.93 ± 0.67	0.048
BUN	33 ± 25	33 ± 26	0.985	19 ± 13	17 ± 11	0.002
PT	17 ± 11	17 ± 12	0.08	13.97 ± 6.52	13.54 ± 5.84	0.154
INR	1.62 ± 1.15	1.62 ± 1.16	0.152	1.26 ± 0.47	1.24 ± 0.77	0.63
Mannitol			0.499			<0.001
No	316 (99.4%)	316 (99.5%)		660 (77.5%)	775 (89.9%)	
Yes	2 (0.6%)	2 (0.7%)		192 (22.5%)	87 (10.1%)	
Norepinephrine			0.002			0.002
No	191 (60.1%)	191 (60.2%)		751 (88.1%)	798 (92.6%)	
Yes	127 (39.9%)	127 (39.10%)		101 (11.9%)	64 (7.4%)	
Vasopressin			0.456			<0.001
No	279 (87.7%)	279 (87.8%)		747 (87.7%)	797 (92.5%)	
Yes	39 (12.3%)	39 (12.4%)		105 (12.3%)	65 (7.5%)	
Invasive ventilator			<0.001			<0.001
No	170 (53.5%)	170 (53.6%)		359 (42.1%)	577 (66.9%)	
Yes	148 (46.5%)	148 (46.6%)		493 (57.9%)	285 (33.1%)	
Surgery			0.366			<0.001
No	317 (99.7%)	317 (99.8%)		664 (77.9%)	736 (85.4%)	
Yes	1 (0.3%)	1 (0.4%)		188 (22.1%)	126 (14.6%)	
IUC.LOS	4.3 ± 5.7	4.3 ± 5.8	<0.001	8 ± 10	6 ± 7	<0.001
Hospital LOS	10 ± 9	10 ± 10	0.022	14 ± 18	12 ± 13	0.003
In-ICU mortality			0.112			<0.001
No	290 (91.2%)	290 (91.3%)		657 (77.1%)	798 (92.6%)	
Yes	28 (8.8%)	28 (8.9%)		195 (22.9%)	64 (7.4%)	
In-hospital mortality.			0.027			<0.001
No	276 (86.8%)	276 (86.9%)		603 (70.8%)	768 (89.1%)	
Yes	42 (13.2%)	42 (13.3%)		249 (29.2%)	94 (10.9%)	
The 30-day mortality			0.116			<0.001
No	267 (84.0%)	267 (84.1%)		589 (69.1%)	737 (85.5%)	
Yes	51 (16.0%)	51 (16.1%)		263 (30.9%)	125 (14.5%)	
The 90-day mortality			0.146			<0.001
No	243 (76.4%)	243 (76.5%)		542 (63.6%)	705 (81.8%)	
Yes	75 (23.6%)	75 (23.7%)		310 (36.4%)	157 (18.2%)	
The 180-day mortality			0.502			<0.001
No	231 (72.6%)	231 (72.7%)		515 (60.4%)	674 (78.2%)	
Yes	87 (27.4%)	87 (27.5%)		337 (39.6%)	188 (21.8%)	
The 1-year mortality			0.534			<0.001
No	215 (67.6%)	215 (67.7%)		485 (56.9%)	641 (74.4%)	
Yes	103 (32.4%)	103 (32.5%)		367 (43.1%)	221 (25.6%)	
Disturbance of consciousness			0.022			<0.001
No	274 (86.2%)	274 (86.3%)		579 (68.0%)	697 (80.9%)	
Yes	44 (13.8%)	44 (13.9%)		273 (32.0%)	165 (19.1%)	
GCS	13.76 ± 2.54	13.76 ± 2.55	<0.001	12.59 ± 3.44	13.22 ± 2.55	<0.001
SOFA	5.4 ± 3.5	5.4 ± 3.6	0.007	3.79 ± 3.16	2.99 ± 2.55	<0.001
APS III	48 ± 21	48 ± 22	0.006	41 ± 20	34 ± 15	<0.001

**Abbreviations**: SBP, systolic blood pressure; DBP, diastolic blood pressure; MBP, mean blood pressure; SpO₂, peripheral oxygen saturation; HF, heart failure; COPD, chronic obstructive pulmonary disease; RF, renal failure; WBC, white blood cell; RBC, red blood cell; HCT, Hematocrit; MCH, Mean Corpuscular Hemoglobin; MCHC, Mean Corpuscular Hemoglobin Concentration; BUN, blood urea nitrogen; PT, Prothrombin Time; INR, International Normalized Ratio; ICU, intensive care unit; GCS, Glasgow Coma Scale; SOFA, Sequential Organ Failure Assessment; SAPS III, Simplified Acute Physiology Score III.

Post-matching, age and sex distributions were well balanced in both hemorrhagic and ischemic stroke subtypes. In hemorrhagic stroke, HG patients had higher glucose levels (mean ± SD: 192.3 ± 70.9 mg/dL) compared to non-HG patients (109.2 ± 18.9 mg/dL), and showed higher rates of comorbidities such as coronary artery disease, heart failure, and renal failure, as well as longer ICU and hospital stays. In ischemic stroke, HG patients also had higher glucose levels (175.5 ± 52.0 mg/dL vs. 111.2 ± 16.9 mg/dL) and more adverse baseline characteristics, including higher prevalence of comorbidities, lower hemoglobin, and elevated creatinine and BUN.

### 3.2. Cox regression models (univariate and multivariate) assessing mortality in stroke patients without documented diabetes

Cox proportional hazards models were applied to assess the association between hyperglycemia (HG) and mortality or severe neurological impairment (GCS < 8) in stroke patients without documented diabetes, both before and after propensity score matching (PSM). Prior to matching, HG was significantly associated with increased short- and long-term mortality in both ischemic stroke (IS) and hemorrhagic stroke (HS) patients (p < 0.001), as well as a higher risk of impaired consciousness (HR = 1.22, *p* = 0.010) (S3–S5 Tables in S1 File).

Following PSM, HG remained an independent predictor of short-term mortality in IS patients, with consistent associations across in-hospital and 30-day outcomes (both *p* < 0.001), although its association with long-term mortality was less stable. In matched IS patients, HG was significantly associated with increased mortality at all time points (*p* < 0.001), reinforcing its prognostic relevance. In contrast, HG was not significantly associated with short-term mortality in HS patients after matching, but was modestly linked to increased 1-year mortality (HR = 0.71, *p* = 0.029), indicating a delayed adverse effect. Notably, HG was no longer significantly associated with neurological impairment after full adjustment (*p* > 0.05), suggesting its effect on functional status may be mediated through other factors (Table 2).

**Table 2:** Cox regression models (univariate and multivariate) assessing mortality in ischemic stroke patients without documented diabetes after PSM.

Characteristic	Model 1			Model 2			Model 3		
	HR^1^	95% CI^1^	p-value	HR^1^	95% CI^1^	p-value	HR^1^	95% CI^1^	p-value
In-hospital mortality									
Non-HG Group	—	—		—	—		—	—	
HG Group	2.55	2.01, 3.24	<0.001	2.51	1.98, 3.19	<0.001	1.69	1.31, 2.17	<0.001
30-day mortality									
Non-HG Group	—	—		—	—		—	—	
HG Group	2.47	2.00, 3.05	<0.001	2.41	1.95, 2.98	<0.001	1.60	1.27, 2.01	<0.001
90-day mortality									
Non-HG Group	—	—		—	—		—	—	
HG Group	2.36	1.95, 2.86	<0.001	2.31	1.91, 2.80	<0.001	1.60	1.30, 1.96	<0.001
180-day mortality									
Non-HG Group	—	—		—	—		—	—	
HG Group	2.17	1.82, 2.59	<0.001	2.13	1.78, 2.54	<0.001	1.54	1.28, 1.87	<0.001
1-year mortality									
Non-HG Group	—	—		—	—		—	—	
HG Group	2.04	1.72, 2.41	<0.001	2.00	1.69, 2.36	<0.001	1.48	1.24, 1.77	<0.001
Consciousness disturbance (GCS < 8)									
Non-HG Group	—	—		—	—		—	—	
HG Group	1.34	1.10, 1.63	0.003	1.33	1.10, 1.62	0.004	1.15	0.93, 1.41	0.196

Model 1: no covariates were adjusted.

Model 2: adjusted for Age and Sex.

Model 3: adjusted for Age, Sex, Respiratory rate, Systolic Blood Pressure, Diastolic Blood Pressure, Mean Blood Pressure, SpO2, Hypertension, Coronary, Heart failure, COPD, Pneumonitis, Renal failure, Kidney disease, Malignancy, WBC count, RBC count, Platelet count, Hemoglobin, HCT, MCHC, Sodium, Potassium, Calcium, Chloride, phosphorus, Creatinine, BUN, PT, INR, Mannitol, Norepinephrine, Vasopressin, Invasive ventilator, Surgery, SOFA, GCS, and APS Ⅲ.

### 3.3. K‒M survival analysis curves

**Fig 2:**
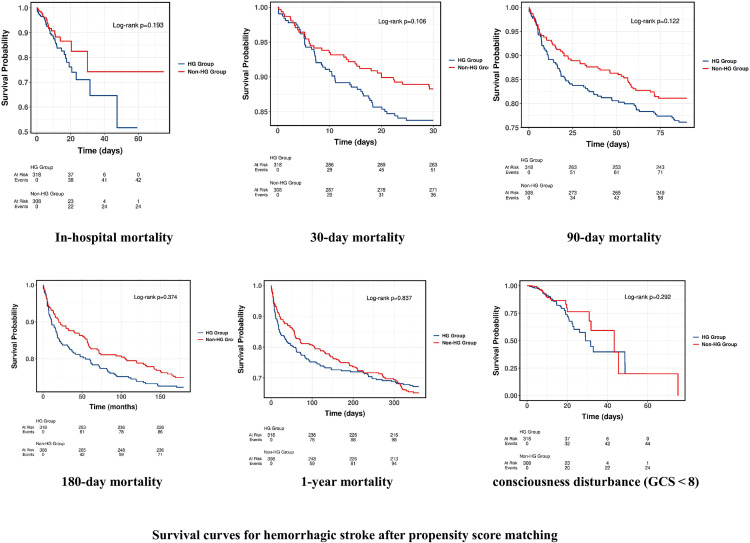
Survival curves for hemorrhagic stroke after propensity score matching. Kaplan-Meier survival curves show survival changes between high risk (HG) and non-high risk (Non-HG) patients with hemorrhagic stroke during hospitalization, 30 days, 90 days, 180 days, 1 year, and in patients with GCS < 8.

**Fig 3:**
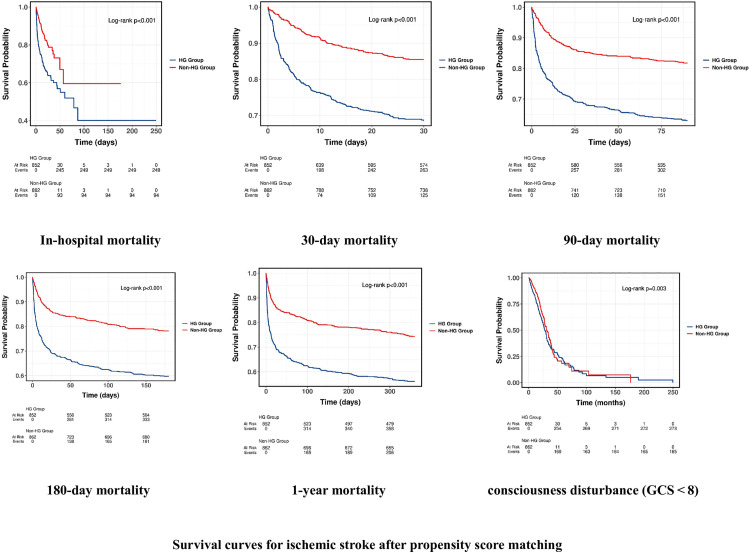
Survival curves for ischemic stroke after propensity score matching. Kaplan-Meier survival curves show survival changes between high risk (HG) and non-high risk (Non-HG) patients with ischemic stroke during hospitalization, 30 days, 90 days, 180 days, 1 year, and in patients with GCS < 8.

Kaplan–Meier survival curves were generated to compare short- and long-term survival between the HG and non-HG groups in stroke patients without documented diabetes, both before and after propensity score matching (PSM). As shown in Fig 2, the HG group exhibited significantly reduced survival at all time points—during hospitalization, and at 30-, 90-, 180-day, and 1-year intervals (log-rank test, all *p* < 0.001). When stratified by stroke subtype, the survival disadvantage in the HG group remained significant among IS patients (Fig 3), whereas in HS patients, only the 1-year mortality was significantly different (*p* = 0.016); no significant differences were observed at earlier time points.

### 3.4. Subgroup analyses

**Fig 4:**
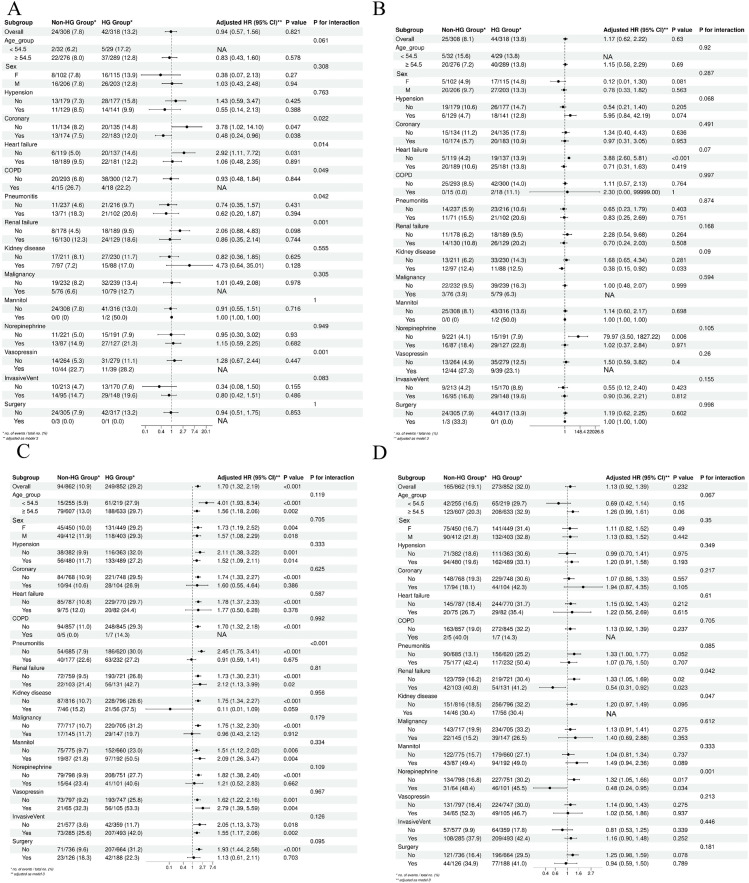
Subgroup analysis. (A) Subgroup analysis for the association of hemorrhagic stroke during hospitalization; (B) Subgroup analysis for the association of hemorrhagic stroke in patients with GCS < 8; (C) Subgroup analysis for the association of ischemic stroke during hospitalization; (D) Subgroup analysis for the association of ischemic stroke in patients with GCS < 8. NA indicates not estimable due to small sample size.

Hyperglycemia had distinct impacts on hemorrhagic stroke (HS) and ischemic stroke (IS) patients, with significant effects observed in specific high-risk subgroups. In HS (Fig 4A, B), hyperglycemia did not significantly affect overall mortality but worsened outcomes in patients with heart failure, pneumonia, vasopressin treatment, and those with severe neurological impairment (GCS < 8) combined with heart failure. In IS (Fig 4C, D), hyperglycemia significantly increased in-hospital mortality, those with age, coronary heart disease, COPD, pneumonia, renal failure, and those receiving norepinephrine treatment. These findings highlight hyperglycemia’s adverse impact, particularly on short-term mortality in high-risk IS patients. These results were derived from stroke-type–stratified analyses to ensure clear comparison.

### 3.5. Sensitivity analysis

To further assess the robustness of the findings, a sensitivity analysis was performed by including all hyperglycemic stroke patients, stratified by the presence of documented diabetes. Across all follow-up periods, patients with documented diabetes consistently showed lower mortality risks compared to those without documented diabetes (adjusted HRs for in-hospital to 360-day mortality ranged from 0.66 to 0.77, all *P *< 0.001). A similar trend was observed for severe consciousness disturbance (adjusted HR = 0.74, 95% CI: 0.63–0.88). Kaplan–Meier curves demonstrated higher survival probabilities in patients with documented diabetes (S8 Tables in S1 File, S4 Fig in S2 File). These results confirm that the main findings remain robust.

## 4. Discussion

This study examined the impact of HG on short-term mortality and consciousness disturbance in stroke patients without documented diabetes, using data from 4,151 individuals in the MIMIC-IV database. HG was consistently associated with higher mortality at all time points—including in-hospital, 30-day, 90-day, 180-day, and 1-year mortality—even after adjustment for demographics, physiological variables, comorbidities, and treatments. This supports its role as an independent prognostic factor.

Subgroup analysis showed that SHG had a strong effect on short-term mortality in ischemic stroke (IS). In contrast, its impact on hemorrhagic stroke (HS) became less significant after multivariable adjustment, possibly due to mediation by factors like hematoma growth or vascular damage. In IS, SHG remained a strong predictor of mortality, particularly in younger patients and those with cardiovascular disease, pneumonia, renal failure, or receiving intensive therapies. In HS patients, SHG was not significantly related to early mortality but was associated with increased 1-year mortality in specific high-risk groups, suggesting a delayed detrimental effect. These subtype-specific trends were derived from stratified analyses, further supporting differential prognostic roles of SHG in IS and HS. The sensitivity analysis including documented diabetic patients confirmed the robustness of the main findings, with consistent trends across mortality endpoints.

Moreover, SHG was linked to increased risk of severe consciousness impairment (GCS < 8) in IS patients, especially those with renal failure or receiving norepinephrine. This supports the hypothesis that hyperglycemia may exacerbate neurological injury through mechanisms such as cerebral edema, blood–brain barrier disruption, and neuroinflammation [20,21].

Impaired consciousness at admission is a well-established marker of stroke severity. In this cohort, reduced GCS was strongly associated with mortality [22]. Neurological injury may directly affect arousal systems or indirectly trigger stress pathways, such as activation of the HPA axis and sympathetic nervous system, resulting in elevated cortisol and catecholamine levels. [23,24]. These hormonal surges promote hyperglycemia by stimulating gluconeogenesis and inducing insulin resistance. Thus, early consciousness disturbance reflects both cerebral injury and systemic stress, underscoring the need for intensive monitoring and proactive management in these patients [25,26].

The hyperglycemia ratio has been shown to exacerbate post-stroke brain injury through various mechanisms, including endothelial dysfunction, oxidative stress, blood-brain barrier disruption, and inflammatory cascades [27,28]. The impact of stress hyperglycemia is particularly pronounced in IS patients [29]. A systematic review of prognostic factors for stroke has demonstrated that, regardless of diabetes status, stress hyperglycemia is associated with worse outcomes. Capes [23] previously highlighted in a systematic review that, among non-diabetic stroke patients, elevated blood glucose levels at admission were significantly correlated with increased in-hospital mortality and poor functional recovery. More recently, a large-scale prospective study in China (ACROSS-China) reported that among non-diabetic IS patients, those in the highest quartile of stress hyperglycemia ratio (measured as the glucose-to-glycated hemoglobin ratio) had a 2- to 3-fold higher risk of stroke recurrence and mortality within one year compared to those in the lowest quartile [30]. Overall, current evidence strongly suggests that stress hyperglycemia in non-diabetic stroke patients is associated with more severe disease progression and worse clinical outcomes. Our findings build upon this understanding by providing additional evidence from a large, ICU-based cohort. In our study, we specifically targeted a patients without documented diabetes population and applied both propensity score matching and multivariable models to reduce confounding, particularly from stroke severity. This allowed us to more clearly establish the independent prognostic impact of stress hyperglycemia. Unlike previous studies that did not clearly separate diabetic and non-diabetic patients, our analysis offers a more refined assessment of the isolated effect of acute stress hyperglycemia on stroke prognosis. Moreover, our analysis extended beyond hospitalization and 30-day outcomes to include 90-day, 180-day, and 1-year mortality, allowing us to capture the evolving impact of stress hyperglycemia on long-term survival. Such long-term follow-up is relatively uncommon in similar studies, enhancing the clinical relevance of our findings by providing valuable insights into the prolonged effects of stress hyperglycemia on stroke outcomes.

Despite yielding meaningful findings, this study has several limitations. First, as a retrospective, single-center study with a relatively limited sample size, our results may be influenced by selection bias and may not be generalizable to broader populations [31,32]. Second, although we excluded patients with a known diagnosis of diabetes based on ICD-coded comorbidities, the absence of uniformly available HbA1c data limited our ability to identify patients with undiagnosed or latent diabetes. This may have led to misclassification bias and attenuation of the observed associations. In addition, hemorrhagic transformation of ischemic stroke was not systematically evaluated, which may also introduce classification bias.

Third, the glycemic exposure was defined based on a single random glucose measurement obtained within 24 hours of ICU admission. While this approach reflects early physiological stress and is consistent with prior MIMIC-based studies, it does not account for glycemic variability, duration of hyperglycemia, or the effects of glucose-lowering interventions [33,34]. Future studies incorporating serial glucose data or continuous glucose monitoring may better capture dynamic glycemic patterns and their prognostic implications.

In addition, while we attempted to define comorbidities—such as renal failure—based on diagnosis codes recorded within 24 hours of ICU admission, the lack of precise diagnosis timestamps in MIMIC-IV limits our ability to completely distinguish pre-existing conditions from early ICU complications.

Fourth, due to the limitations of the MIMIC-IV database, we were unable to access validated neurological outcome scales such as the modified Rankin Scale (mRS) or NIH Stroke Scale (NIHSS). Although we used the Glasgow Coma Scale (GCS) as a surrogate marker of neurological impairment, it primarily reflects acute consciousness levels and does not fully capture long-term functional outcomes.

Moreover, this study was specifically designed to evaluate stress hyperglycemia in patients without documented diabetes, and comparisons with diabetic individuals were not conducted. Future research should explore how baseline metabolic status may modify the impact of acute glycemic disturbances on outcomes. Lastly, although we applied propensity score matching and multivariate adjustment to reduce confounding, the influence of unmeasured or unknown variables cannot be fully excluded. We also acknowledge that our analysis did not include stratification by hyperglycemia severity, which may have limited our ability to explore potential dose-response relationships. This remains an important avenue for future investigation.

In view of the above limitations, we propose the following optimization suggestions. First, large-scale, multicenter prospective studies involving diverse populations are needed to enhance generalizability and minimize selection bias [35]. Second, dynamic glucose monitoring—through continuous or serial measurements—should be applied during the acute phase to better capture the duration and severity of hyperglycemia and their prognostic relevance. Interventional studies targeting glycemic control could further explore whether active management of stress hyperglycemia improves outcomes and supports causal inference.

Future studies should also include functional outcome measures—such as neurological recovery and disability—and adopt more comprehensive assessments of stroke severity, including NIHSS scores [36], imaging-based infarct or hemorrhage volume [37], and lesion location (particularly hypothalamic or autonomic centers) [38], to better understand how these factors influence glycemic response. Lastly, mechanistic studies investigating stress hormones (cortisol, catecholamines) and inflammatory markers in acute stroke may help elucidate the biological pathways linking stress hyperglycemia to prognosis. Such refinements may generate more robust evidence and contribute to the development of personalized management strategies for stroke patients.

## 5. Conclusions

This study identified stress hyperglycemia as an independent risk factor for poor short-term and long-term prognosis in stroke patients without documented diabetes, especially in patients with IS. An in-depth analysis of patients with HS and long-term prognosis assessment found that stress hyperglycemia may have a weak effect on short-term mortality in patients with HS, but still has an effect on long-term survival. Studies have also suggested that disturbance of consciousness in the context of stress hyperglycemia may further aggravate the progress of stroke patients. In addition, this research progress provides a more specific basis for clinical blood glucose management strategies, with special attention to individualized intervention strategies in different stroke subtypes, suggesting that personalized treatment programs for stroke patients can be further optimized in the future.

## Supporting information

S1 File S1-S8 Tables. (XLSX)

S2 File S1-S4 Figures. (DOCX)

## Abbreviation

BBB: blood-brain barrier
IS: ischemic stroke
HS: hemorrhagic stroke
BIDMC: Beth Israel Deaconess Medical Center
IRB: Institutional Review Board
CITI: Collaborative Institutional Training Initiative
ICU: intensive care unit
GCS: Glasgow Coma Scale
SQL: Structured Query Language
RCS: Restricted cubic spline
non-HG: non-hyperglycemia group
HG: hyperglycemia group
PSM: Propensity score matching
COPD: chronic obstructive pulmonary disease
WBC: white blood cell
RBC: red blood cell
HCT: hematocrit
MCHC: mean corpuscular hemoglobin concentration
BUN: blood urea nitrogen
PT: prothrombin time
INR: international normalized ratio
HRs: hazard ratios
CIs: confidence intervals
SOFA: Sequential Organ Failure Assessment
APS III: Acute Physiology Score III
ASD: absolute standardized differences
HPA: hypothalamic-pituitary-adrenal

## References

[1] Roth GA, Mensah GA, Johnson CO, Addolorato G, Ammirati E, Baddour LM, et al. Global burden of cardiovascular diseases and risk factors, 1990-2019: update from the GBD 2019 study. J Am Coll Cardiol. 2020;76(25):2982–3021. doi: 10.1016/j.jacc.2020.11.010 33309175 PMC7755038

[2] Zhang H, Zhan Q, Dong F, Gao X, Zeng F, Yao J, et al. Associations of Chinese visceral adiposity index and new-onset stroke in middle-aged and older Chinese adults: an observational study. Lipids Health Dis. 2023;22(1):74. doi: 10.1186/s12944-023-01843-x 37337187 PMC10280837

[3] Campbell BCV, Khatri P. Stroke. Lancet. 2020;396(10244):129–42. doi: 10.1016/S0140-6736(20)31179-X 32653056

[4] Hilkens NA, Casolla B, Leung TW, de Leeuw F-E. Stroke. Lancet. 2024;403(10446):2820–36. doi: 10.1016/S0140-6736(24)00642-1 38759664

[5] Williams LS, Rotich J, Qi R, Fineberg N, Espay A, Bruno A, et al. Effects of admission hyperglycemia on mortality and costs in acute ischemic stroke. Neurology. 2002;59(1):67–71. doi: 10.1212/wnl.59.1.67 12105309

[6] Zhang H, Jiang X, Li A, Wang X. Causal Associations Between Gut Microbiota and Cerebrovascular Diseases. World Neurosurg. 2024;183:e587–97. doi: 10.1016/j.wneu.2023.12.150 38191059

[7] Snarska KK, Bachórzewska-Gajewska H, Kapica-Topczewska K, Drozdowski W, Chorąży M, Kułakowska A, et al. Hyperglycemia and diabetes have different impacts on outcome of ischemic and hemorrhagic stroke. Arch Med Sci. 2017;13(1):100–8. doi: 10.5114/aoms.2016.61009 28144261 PMC5206364

[8] Mifsud S, Schembri EL, Gruppetta M. Stress-induced hyperglycaemia. Br J Hosp Med (Lond). 2018;79(11):634–9. doi: 10.12968/hmed.2018.79.11.634 30418830

[9] Newsholme P, Cruzat VF, Keane KN, Carlessi R, de Bittencourt PIH Jr. Molecular mechanisms of ROS production and oxidative stress in diabetes. Biochem J. 2016;473(24):4527–50. doi: 10.1042/BCJ20160503C 27941030

[10] Broocks G, Kemmling A, Aberle J, Kniep H, Bechstein M, Flottmann F, et al. Elevated blood glucose is associated with aggravated brain edema in acute stroke. J Neurol. 2020;267(2):440–8. doi: 10.1007/s00415-019-09601-9 31667625

[11] Zhang F, Zhang S, Tao C, Yang Z, Li X, You C, et al. Association between serum glucose level and spot sign in intracerebral hemorrhage. Medicine (Baltimore). 2019;98(11):e14748. doi: 10.1097/MD.0000000000014748 30882643 PMC6426545

[12] Lin L, Yang J, Chen C, Tian H, Bivard A, Spratt NJ, et al. Association of Collateral Status and Ischemic Core Growth in Patients With Acute Ischemic Stroke. Neurology. 2021;96(2):e161–70. doi: 10.1212/WNL.0000000000011258 33262233

[13] McCall SJ, Alanazi TA, Clark AB, Musgrave SD, Bettencourt-Silva JH, Bachmann MO, et al. Hyperglycaemia and the SOAR stroke score in predicting mortality. Diab Vasc Dis Res. 2018;15(2):114–21. doi: 10.1177/1479164117743034 29185347

[14] Zhang H, Jiao L, Yang S, Li H, Jiang X, Feng J, et al. Brain-computer interfaces: the innovative key to unlocking neurological conditions. Int J Surg. 2024;110(9):5745–62. doi: 10.1097/JS9.0000000000002022 39166947 PMC11392146

[15] Xie L, Chen J, Li Y, Huang B, Luo S. The prognostic impact of stress hyperglycemia ratio on mortality in cardiogenic shock: a MIMIC-IV database analysis. Diabetol Metab Syndr. 2024;16(1):312. doi: 10.1186/s13098-024-01562-y 39719644 PMC11667900

[16] Huang Y-W, Zhang Y, Li Z-P, Yin X-S. Association between a four-parameter inflammatory index and all-cause mortality in critical ill patients with non-traumatic subarachnoid hemorrhage: a retrospective analysis of the MIMIC-IV database (2012-2019). Front Immunol. 2023;14:1235266. doi: 10.3389/fimmu.2023.1235266 37936706 PMC10626529

[17] Tanaka K, Yoshimoto T, Koge J, Yamagami H, Imamura H, Sakai N, et al. Detrimental Effect of Acute Hyperglycemia on the Outcomes of Large Ischemic Region Stroke. J Am Heart Assoc. 2024;13(23):e034556. doi: 10.1161/JAHA.124.034556 39575760 PMC11681584

[18] American Diabetes Association. 15. Diabetes Care in the Hospital: Standards of Medical Care in Diabetes-2020. Diabetes Care. 2020;43(Suppl 1):S193–202. doi: 10.2337/dc20-S015 31862758

[19] Thomas LE, Thomas SM, Li F, Matsouaka RA. Addressing substantial covariate imbalance with propensity score stratification and balancing weights: connections and recommendations. Epidemiol Methods. 2023;12(1):20220131. doi: 10.1515/em-2022-0131 38013684 PMC10637625

[20] Jiang X, Andjelkovic AV, Zhu L, Yang T, Bennett MVL, Chen J, et al. Blood-brain barrier dysfunction and recovery after ischemic stroke. Prog Neurobiol. 2018;163–164:144–71. doi: 10.1016/j.pneurobio.2017.10.001 28987927 PMC5886838

[21] Yang C, Hawkins KE, Doré S, Candelario-Jalil E. Neuroinflammatory mechanisms of blood-brain barrier damage in ischemic stroke. Am J Physiol Cell Physiol. 2019;316(2):C135–53. doi: 10.1152/ajpcell.00136.2018 30379577 PMC6397344

[22] Healy RJ, Zorrilla-Vaca A, Ziai W, Mirski MA, Hogue CW, Geocadin R, et al. Glasgow Coma Scale Score Fluctuations are Inversely Associated With a NIRS-based Index of Cerebral Autoregulation in Acutely Comatose Patients. J Neurosurg Anesthesiol. 2019;31(3):306–10. doi: 10.1097/ANA.0000000000000513 29782388 PMC6240506

[23] Capes SE, Hunt D, Malmberg K, Pathak P, Gerstein HC. Stress hyperglycemia and prognosis of stroke in nondiabetic and diabetic patients: a systematic overview. Stroke. 2001;32(10):2426–32. doi: 10.1161/hs1001.096194 11588337

[24] Tsai Y-H, Yuan R, Huang Y-C, Yeh M-Y, Lin C-P, Biswal BB. Disruption of brain connectivity in acute stroke patients with early impairment in consciousness. Front Psychol. 2014;4:956. doi: 10.3389/fpsyg.2013.00956 24427147 PMC3877750

[25] Christensen H, Boysen G, Johannesen HH. Serum-cortisol reflects severity and mortality in acute stroke. J Neurol Sci. 2004;217(2):175–80. doi: 10.1016/j.jns.2003.09.013 14706221

[26] Duan H, Yun HJ, Rajah GB, Che F, Wang Y, Liu J, et al. Large vessel occlusion stroke outcomes in diabetic vs. non-diabetic patients with acute stress hyperglycemia. Front Neurosci. 2023;17:1073924. doi: 10.3389/fnins.2023.1073924 36777640 PMC9911880

[27] Guo X, Li H, Zhang Z, Li S, Zhang L, Zhang J, et al. Hyperglycemia and mortality risk in patients with primary intracerebral hemorrhage: a meta-analysis. Mol Neurobiol. 2016;53(4):2269–75. doi: 10.1007/s12035-015-9184-4 25972238

[28] Jiang Z, Wang K, Duan H, Du H, Gao S, Chen J, et al. Association between stress hyperglycemia ratio and prognosis in acute ischemic stroke: a systematic review and meta-analysis. BMC Neurol. 2024;24(1):13. doi: 10.1186/s12883-023-03519-6 38166660 PMC10759321

[29] Gilotra K, Basem J, Janssen M, Swarna S, Mani R, Ren B, et al. Stress-Induced Hyperglycemia Predicts Poor Outcomes in Primary Intracerebral Hemorrhage Patients. NeuroSci. 2025;6(1):12. doi: 10.3390/neurosci6010012 39982264 PMC11843840

[30] Zhu B, Pan Y, Jing J, Meng X, Zhao X, Liu L, et al. Stress Hyperglycemia and Outcome of Non-diabetic Patients After Acute Ischemic Stroke. Front Neurol. 2019;10:1003. doi: 10.3389/fneur.2019.01003 31620074 PMC6759951

[31] Thygesen LC, Ersbøll AK. When the entire population is the sample: strengths and limitations in register-based epidemiology. Eur J Epidemiol. 2014;29(8):551–8. doi: 10.1007/s10654-013-9873-0 24407880

[32] Odhav A, Belsito DV. Is quaternium-15 a formaldehyde releaser? Correlation between positive patch test reactions to formaldehyde and quaternium-15. Dermatitis. 2012;23(1):39–43. doi: 10.1097/DER.0b013e31823d1785 22653068

[33] Sartore G, Chilelli NC, Burlina S, Di Stefano P, Piarulli F, Fedele D, et al. The importance of HbA1c and glucose variability in patients with type 1 and type 2 diabetes: outcome of continuous glucose monitoring (CGM). Acta Diabetol. 2012;49 Suppl 1:S153-60. doi: 10.1007/s00592-012-0391-4 22466072

[34] Kovatchev BP. Metrics for glycaemic control - from HbA1c to continuous glucose monitoring. Nat Rev Endocrinol. 2017;13(7):425–36. doi: 10.1038/nrendo.2017.3 28304392

[35] Zhang H, Chen Y, Jiang X, Gu Q, Yao J, Wang X, et al. Unveiling the landscape of cytokine research in glioma immunotherapy: a scientometrics analysis. Front Pharmacol. 2024;14:1333124. doi: 10.3389/fphar.2023.1333124 38259287 PMC10800575

[36] Helenius J, Henninger N. Leukoaraiosis Burden Significantly Modulates the Association Between Infarct Volume and National Institutes of Health Stroke Scale in Ischemic Stroke. Stroke. 2015;46(7):1857–63. doi: 10.1161/STROKEAHA.115.009258 25999386

[37] Rosenberg NF, Liebling SM, Kosteva AR, Maas MB, Prabhakaran S, Naidech AM. Infarct volume predicts delayed recovery in patients with subarachnoid hemorrhage and severe neurological deficits. Neurocrit Care. 2013;19(3):293–8. doi: 10.1007/s12028-013-9869-3 23860664

[38] Geest V, Oblak JP, Popović KŠ, Nawabi J, Elsayed S, Friedrich C, et al. How much of the variance in functional outcome related to intracerebral hemorrhage volume is already apparent in neurological status at admission?. J Neurol. 2024;271(8):5003–11. doi: 10.1007/s00415-024-12427-9 38775933 PMC11319529

