# Supplementary material for: The influence of stress hyperglycemia on consciousness disturbance and short- and long-term outcomes in stroke patients without documented diabetes: Differences between ischemic and hemorrhagic stroke
Source: PLoS One. 2025 Aug 26;20(8):e0331077. doi: 10.1371/journal.pone.0331077 (PMC12380300; doi:10.1371/journal.pone.0331077)
Supplement: S2 File — (DOCX) [file pone.0331077.s002.docx]

**S1 Figure**

**
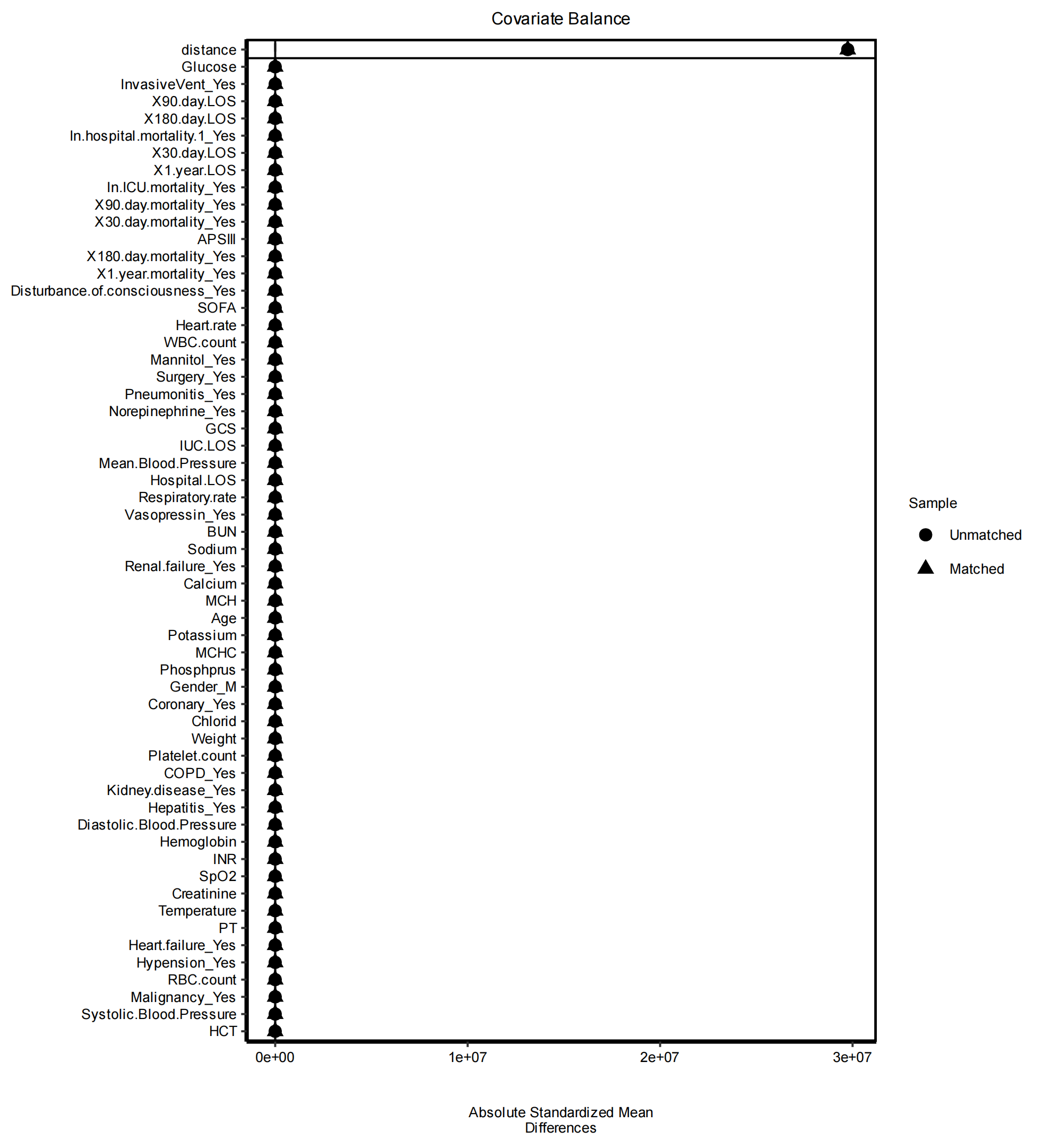
**

**S1 Figure.** The absolute standardized differences for variables used to match the two groups.

**S2 Figure**

**
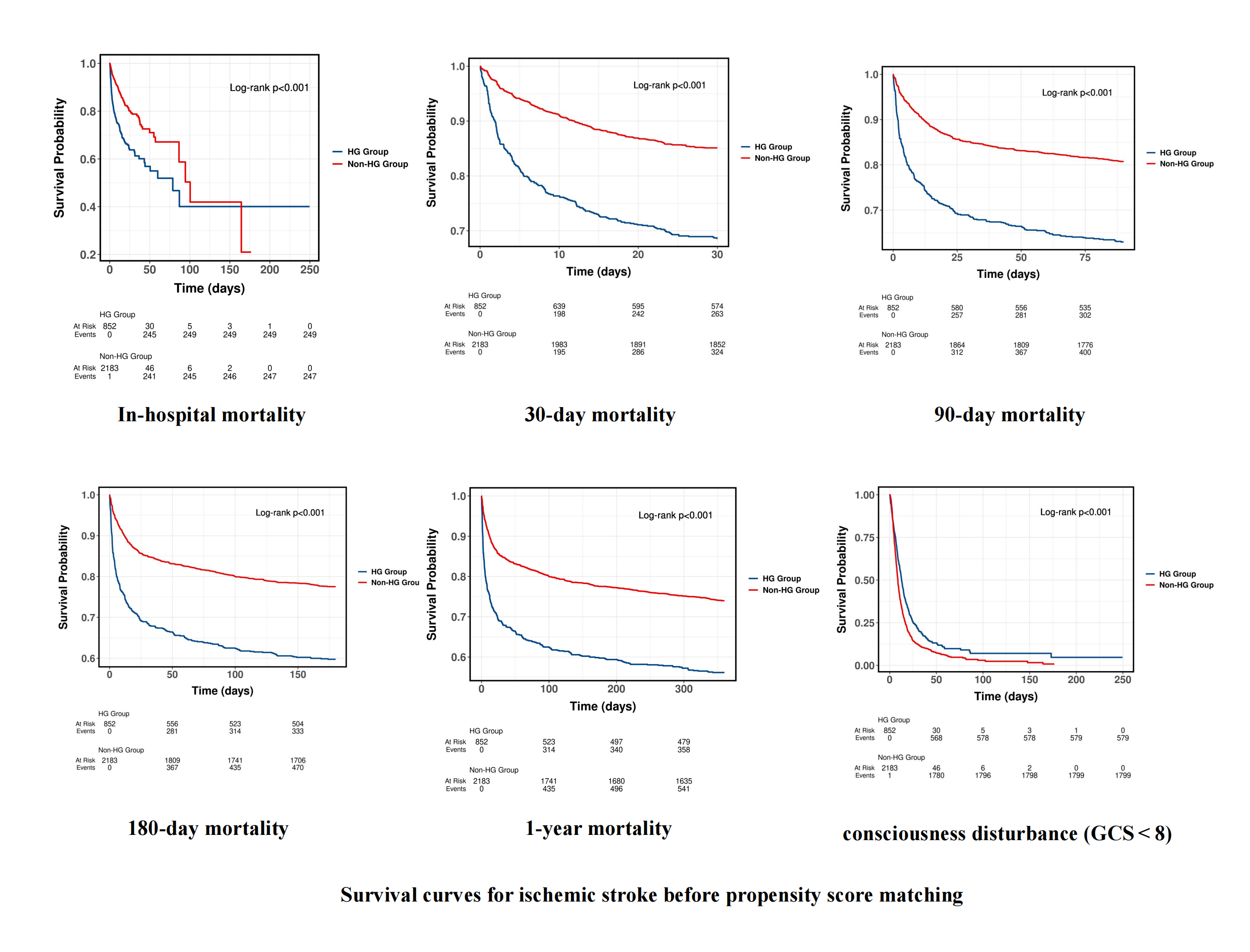
**

**S2 Figure**: **Survival curves for ischemic stroke before propensity score matching**

Kaplan-Meier survival curves show the changes in survival between high risk (HG) and non-high risk (Non-HG) ischemic stroke patients in hospital, 30 days, 90 days, 180 days, 1 year, and patients with GCS<8.

**S3 Figure**

**
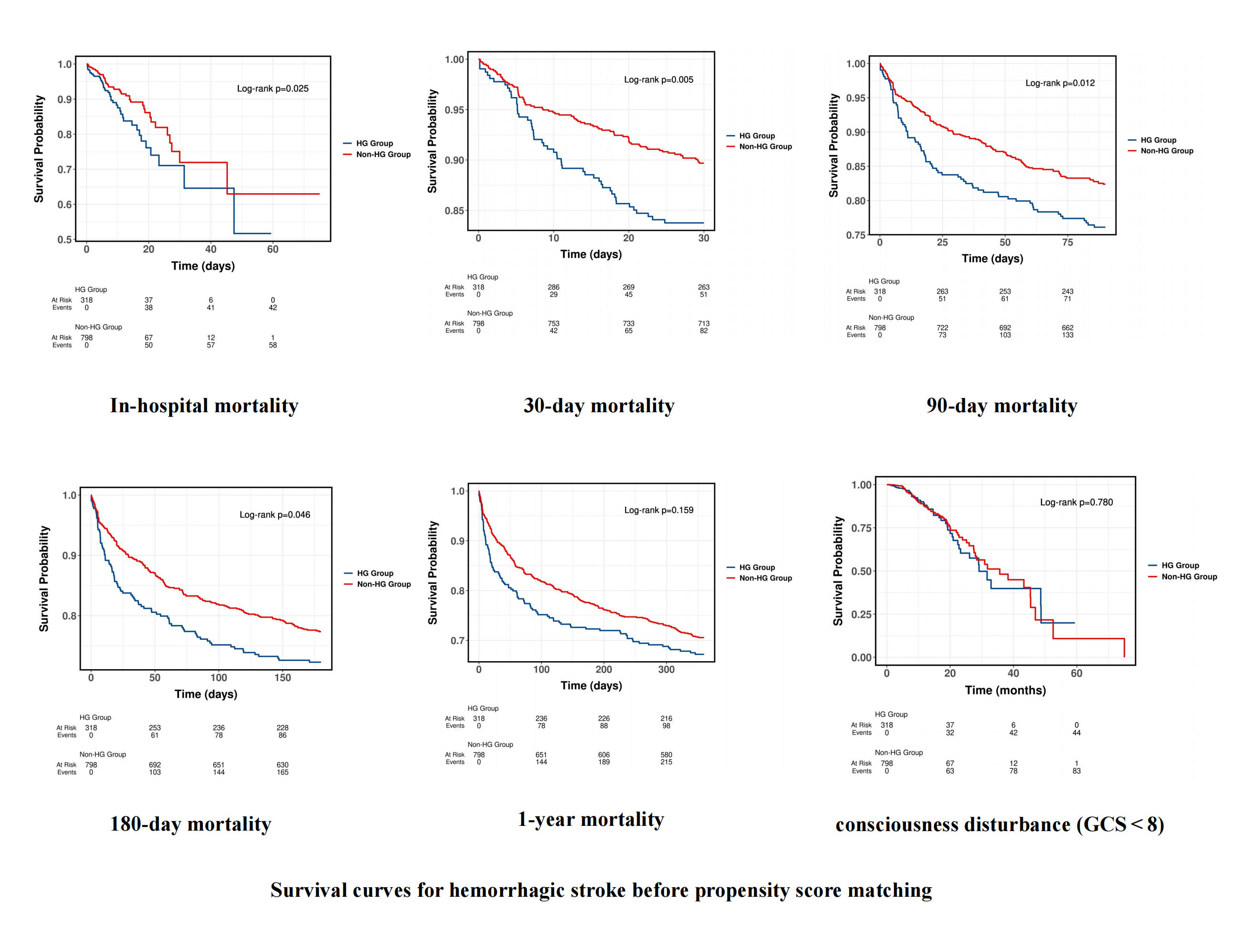
**

**S3 Figure: Survival curves for hemorrhagic stroke before propensity score matching**

Kaplan-Meier survival curves show survival changes between high risk (HG) and non-high risk (Non-HG) patients with non-diabetic hemorrhagic stroke during hospitalization, 30 days, 90 days, 180 days, 1 year, and in patients with GCS<8.

**S4 Figure**


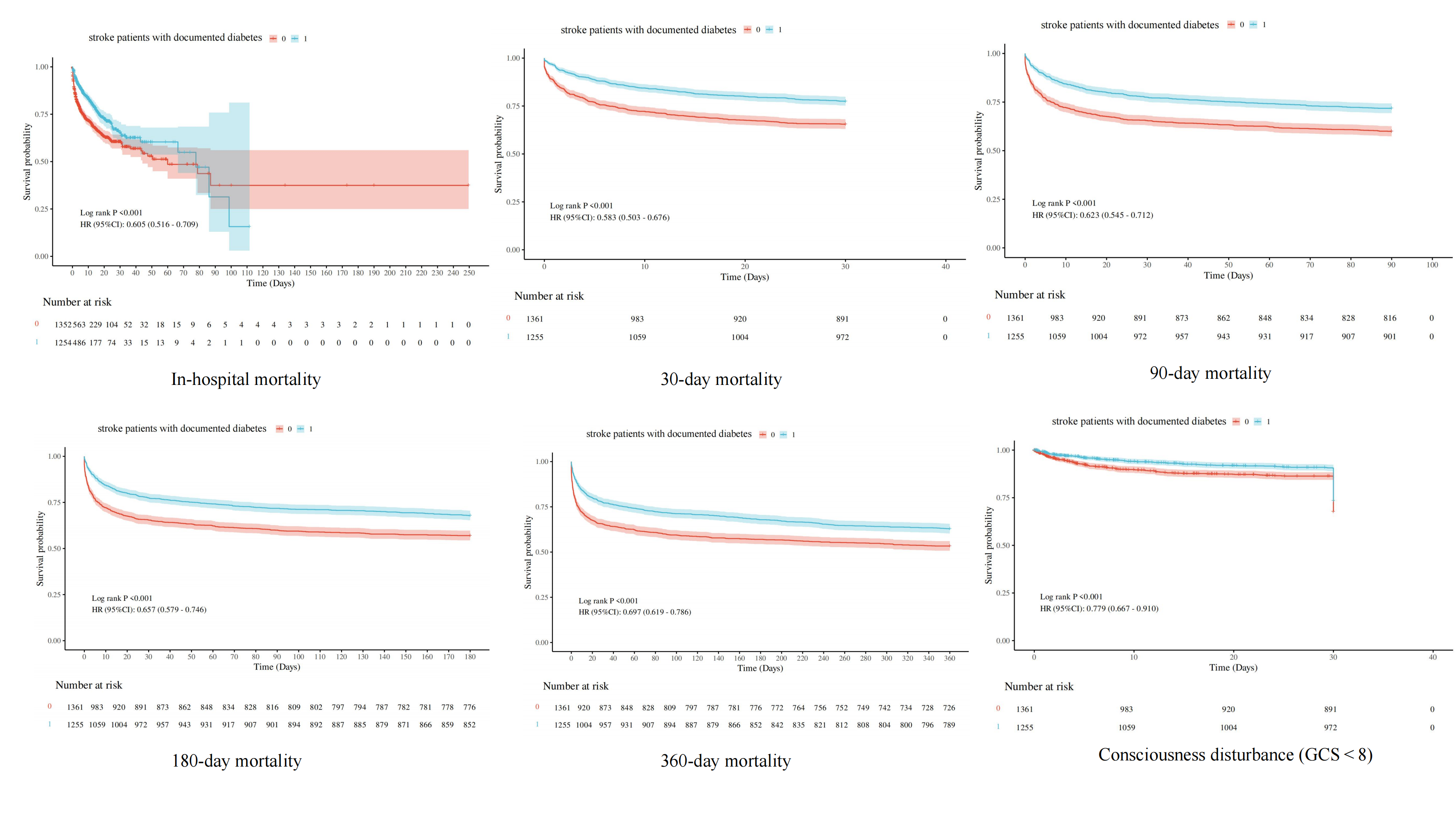


**S4 Figure Kaplan–Meier survival curves for hyperglycemic stroke patients stratified by documented diabetes status.**

Kaplan–Meier survival analysis comparing hyperglycemic stroke patients with documented diabetes (blue curves) and without documented diabetes (red curves) for in-hospital, 30-day, 90-day, 180-day, and 360-day mortality, as well as severe consciousness disturbance (GCS <8). Shaded areas represent 95% confidence intervals.
